# Using fuzzy-set qualitative comparative analysis

**DOI:** 10.4178/epih/e2014038

**Published:** 2014-12-31

**Authors:** Sophia Seung-yoon Lee

**Affiliations:** Department of Social Welfare, Ewha Womans University, Seoul, Korea

Fuzzy-set qualitative comparative analysis (Fs/QCA) is a social science method developed in order to combine case-oriented and variable-oriented quantitative analysis. It started with the creation of qualitative comparative analysis [1], with Fs/QCA later developed by applying fuzzy-set theory [2]. Although the fuzzy-set approach is still considered to be in the process of being developed [3-5], it has already been discussed as a useful methodology among social scientists. In addition, the number of researchers across disciplines who use this methodology has steadily increased.

Fs/QCA recognizes the limitation of case-oriented research in theorization and scientific measurement. In most cases, researchers begin their case-oriented research based upon theoretical concepts and then select cases to refine and elaborate on these concepts. In Fs/QCA, a set membership is scientifically calibrated by using empirical evidence. At the same time, a set-theoretic approach is incorporated to increase the connectivity between theories and methodologies, a limitation of variable-oriented research. In other words, case-oriented research has acquired some of the characteristics of variable-oriented research through calibration as well as retaining its own characteristics with the use of set theory.

The first main characteristic of Fs/QCA is the degrees of freedom allowed for researchers to examine the causal complexities involved in conjunctural causation [2]. The fundamental aim of case-oriented comparative analysis is to examine cases configurationally. Because separate parts consisting of the whole are considered to be understood in the whole picture or configurationally in case-oriented analysis [2], researchers are concerned with understanding the context of a case. In addition, with the use of various theories, they look into various aspects of the case to obtain a comprehensive understanding of the homogeneity within it.

Similarly, Fs/QCA focuses on a joint causal system that allows interaction effects among each characteristic within a case rather than a methodology in which the independent variables are considered constant and the effects of a cause on the dependent variables are examined. For example, in Fs/QCA, the independent effects of the health care system and democracy are not verified as a causal condition of “citizens’ health level.” On the contrary, four causal conditions are constructed as a configuration and the relationship between configurational conditions and health levels are analyzed. In other words, when the existence of the health care system (here, divided into low and high levels of health care systems) and democracy (here, divided into highly developed and underdeveloped democracies) are chosen as the hypothetical conditions accountable for citizens’ health level, each independent variable’s effect on citizens’ health level (the dependent variable) is not analyzed, rather how each condition is combined and how it functions as a factor are assessed.

In Fs/QCA, causal conditions are explained as “attributes.” When there are K attributes, up to 2^k^ causal combinations can be constructed mathematically (H means a high level of health care system, h a low level of health care system, D a highly developed democracy, and d an underdeveloped democracy).

X1 = high level of health care system with a highly developed democracy (HD),

X2 = high level of health care system with an underdeveloped democracy (Hd),

X3 = low level of health care system with a highly developed democracy (hD),

X4 = low level of health care system with an underdeveloped democracy (hd).

An equation explaining the causal relations can then be developed by applying “logical and” and “logical or” to cases based upon qualitative research. The “logical and” indicates a compound set combined with more than two conditions. It is written as ^*^ in the equation. The “logical or” means a sum of sets and this is written as + in the equation.

Let us suppose that researchers choose 12 cases (countries) based upon a theory, eight of which have citizens with a high level of health. Let us further suppose that researchers analyze these eight cases and find three applicable causal configurations: 1) a high level of health care system with a highly developed democracy (HD), 2) a high level of health care system with an underdeveloped democracy (Hd), and 3) a low level of health care system with a highly developed democracy (hD). This means that three configurations may be configurational conditions for citizens’ high level of health. In other words, HD, Hd, or hD can constitute the causal conditions for healthy citizens. Thus,

Citizens’ high level of health=HD+Hd+hD

When the Boolean approach is applied, the above equation becomes as simple as the following. According to the following equation, if “a high level of health care system” or “a low level of health care system with a highly developed democracy (H+ hD)” exists, a citizen’s health level is high. In other words, if a high level of health care system exists, it can constitute a condition for citizens’ high level of health regardless of the level of democratic development. In addition, even if a low level of health care exists, a highly developed democracy can constitute a condition for a high level of health. Thus,

Citizens’ high level of health=HD+Hd+hD=H (D+d)+hD=H+hD

Secondly, Fs/QCA emphasizes cases more than variables. In a conventional variable-oriented analysis, when there is a pooling of many cases, subtle historical or contextual differences within the cases are likely to be ignored because of the lack of specificity within them. In other words, heterogeneity within the cases can be easily overlooked. The advantage of Fs/QCA is that each case is not dissected into variables but analyzed as a whole. The membership scores of each configuration (e.g., the membership scores for HD, Hd, or hD) for cases as a whole can be created.

Thirdly, Fs/QCA allows flexibility in the characterization of cases. As mentioned above, each case is assigned membership scores for the important causal conditions such as the health care system and level of democracy. Whereas set theory allows a case to be either in a set or out of a set, fuzzy-set theory allows degrees of membership to a case. Fs/QCA accepts degrees of difference in attributes within a case, too. These degrees of membership are determined by researchers. For example, if the health care system in the UK is divided into a dichotomy, such as “fully in” or “fully out,” based upon conventional set theory, by combining with fuzzy-set theory, each condition is assigned by researchers membership scores such as degree of development in the health care system or government expenditure based upon the research purposes. Researchers define the break point, such as 1 (fully in) and 0 (fully out), and decide the membership scores from 0 to 1 indicating partial membership. In other words, membership score is a fine-grained, continuous measure calibrated by researchers by utilizing fundamental and theoretical knowledge related to the attributes of the conditions. This calibration allows the quantitative comparison of qualitative concepts. For example, when researchers decide whether a country should be assigned as democratic or not, or when they compare countries based upon citizens’ health level, they calibrate the membership scores by using their theoretical and qualitative knowledge. Through this, the quantitative comparison of a qualitative concept such as health level is enabled. In Fs/QCA, calibration (i.e., deciding on the degree of membership) can vary based upon different concepts. Hence, it reflects conceptual, theoretical, historical, and contextual backgrounds based upon researchers’ priorities.

These characteristics of Fs/QCA have two advantages. Firstly, Fs/QCA reflects the general processes of a qualitative analysis adequately. Secondly, careful calibration allows quantitative analysis. Thirdly, Fs/QCA enables theories to coincide with empirical data analyses by applying the set-theoretic characteristics embedded in various propositions of social science [2]. In fuzzy-set analysis, calibration is a membership scoring scheme. This measure developed by researchers enables them to interpret the scores directly. For example, in natural science, 20°C is interpretable because it is situated between 0°C and 100°C. Also, in theoretical narratives such as “the development of democracy is a necessity for citizens’ high level of health,” citizens’ high level of health constitutes a subset of democracy. That is to say, democracy is a necessary condition for citizens’ health. Ragin explained that set relations are units consisting of linguistic narratives and in quantitative studies, set relations described in theories are mostly converted into correlational hypotheses between variables, centering on the net-effects evaluation of the causal variables. Therefore, they shift away from theoretical discourse.

The conditions, postulated as causal factors (Xi) or membership scores to uncover the causal relations in set theory, need to be measured in the same way as the membership scores of the outcome (Yi) [2]. If the membership scores of one condition are consistently more than or equal to those of the outcome, this is a necessary condition (Yi≤Xi). If the membership scores of one come, this is a sufficient condition (Xi≤Yi). To find out whether one condition is a sufficient or necessary condition, researchers should calculate the membership scores of conditions such as H (a high level of health care system) and D (a highly developed democracy) for each case. If the membership scores of H and D are consistently less than those of citizens’ health level, these conditions are sufficient.

For example, eight countries, whose citizens are evaluated to have a high level of health, have different membership scores for H and D. If Singapore has 0.7 for H, 0.5 for D, and 0.8 for C (citizens’ health level), the maximum score for the causal relations is 0.7. Since this is lower than the score of the outcome, which is 0.8, the case of Singapore supports the following argument that a high level of health care system or “a low level of health care system with a highly developed democracy (H+hD) is a sufficient condition for citizens’ high level of health. If all of the eight countries show a consistent outcome (Xi≤Yi), a high level of health care system or a low level of health care system with a highly developed democracy (H+hD) is a sufficient condition for citizens’ high level of health.

The basic steps for the foregoing Fs/QCA can be summarized into the following five steps. (1) The characteristics or causal conditions of ideal types are selected based upon qualitative analysis and knowledge of existing theory on a case. (2) The available configurative conditions are presented, which can be simplified by using the Boolean approach. (3) A membership scoring scheme (calibration) is constructed from empirical knowledge and related theories of the case. Based upon this, break points are determined by the researchers. (4) Membership scores for each condition or configurative membership scores for each case are calculated. (5) Membership scores for the causal conditions and outcome are calculated and compared to find the necessary and sufficient conditions [6].

In summary, Fs/QCA is a methodology that aims to include the advantages of both qualitative and quantitative analyses. When well executed, it can bridge the gap between these two methodologies. However, it is of foremost importance for researchers to have a firm grasp of the unique functions of Fs/QCA and apply them to research appropriately.
