# Supplementary material for: Using fuzzy-set qualitative comparative analysis
Source: Epidemiol Health. 2014 Dec 31;36:e2014038. doi: 10.4178/epih/e2014038 (PMC4322522; doi:10.4178/epih/e2014038)
Supplement: Supplementary file 1 [file epih-36-e2014038-supplementary.pdf]

Epidemiology & Health(epiH) – Invited Lecture

퍼지셋 질적비교연구방법론의 활용 (Using Fuzzy-set Qualitative analysis)

이승윤

이화여자대학교 (sophia.sy.lee@ewha.ac.kr)

퍼지셋 질적비교연구(Fs/QCA)는 사회과학 방법론에서 사례중심의 질적분석과 변수중심의 양적분석을 통합하기 위해 개발된 시도로 먼저 질적비교연구(Qualitative Comparative Analysis)의 개발로 시작되었고(Ragin 1987), 이후 여기에 퍼지셋 논리를 적용한 퍼지셋 질적비교연구 (Fs/QCA)가 개발되었다(Ragin 2000). 퍼지셋 방법론적 접근은 아직 상대적으로 발전 과정에 있다고 볼 수 있지만(Ragin 2006; Smithson 2005; Verkuilen 2005), 이미 많은 사회과학자들 사이에서 방법론적 논의의 대상이 되고 있으며 본 방법론을 활용하는 연구자들도 다양한 학문 영역에서 꾸준히 증가하고 있다.

퍼지셋 질적비교연구(Fs/QCA)는 사례중심 연구(case oriented research)가 이론화 및 과학적 측정에 있어 갖게 되는 한계를 인식한다. 사례 중심 연구자는 대개 이론적 틀에서 대상이 되는 개념을 중심으로 연구를 시작하고 이 개념을 정제하고 정교화하기 위하여 선별된 사례들을 활용한다. 퍼지셋 질적비교연구(Fs/QCA)는 경험적 증거를 통한 과학성의 확보를 위해 집합소속 정도(set membership)에 대한 눈금매기기(calibration)를 실시한다. 그러면서도 동시에 변수중심의 연구가 갖는 한계로 지적되는 방법론과 이론과의 연결성을 높이기 위해 집합 이론적 접근(set theoretic approach)을 시도한다. 즉, 눈금매기기를 통해 변수중심 연구의 속성을 가지면서도 집합이론을 활용하여 사례중심 연구의 속성도 내포하고 있다고 볼 수 있는 것이다.

퍼지셋 질적비교연구방법론의 주요 특징으로 첫째, 퍼지셋 질적비교연구방법은 양적 분석과 비교할 때, 결합된 요인들의 인과관계(conjunctural causation)가 내포하는 인과적 복합성(casual complexities)을 살펴보기 위하여 연구자에게 더 많은 자유도(degrees of freedom)를 허용한다(Ragin 2000). 사례중심 비교연구의 기본 논리는 사례를 결합된 총체 중심으로(configurationally) 살펴보는 것이다. 사례중심 분석은 총체를 이루는 서로 다른 개별 부분들이 전체적 그림 혹은 총체적 관점에서 이해된다는 것을 가정하기 때문에 (Ragin 2000), 사례중심 연구자들은 사례가 갖는 맥락을 이해하는 것을 중시한다. 또한 사례 내의 동질성을 포괄적으로 이해하기 위하여 연구자들은 다양한 이론을 활용하여 사례의 여러 측면을 살펴본다.

이와 비슷하게 퍼지셋 질적비교연구(Fs/QCA)는 다른 독립변수들이 일정하다고 간주하고 한 원인이 종속변수에 미치는 영향을 고려하는 방법보다는 사례 내 각각의 특성 간 상호작용 효과를 완전히 허용하는 결합적 인과성 구조(joint causal system)에 초점을 둔다. 예를 들어, 퍼지셋 질적비교연구(Fs/QCA)는 '국민의 건강수준'에 대한 원인조건으로 의료보장제도(health care system)와 민주주의(democracy)의 독립적 효과를 검증하는 것이 아니라, 네 가지의 인과적 결합(configurative) 조건을 만들어 내고, 결합된 조건들(configurational conditions)과 건강수준의 관계를 분석한다. 즉, 의료보장제도의 유무(이 경우 성숙한 의료보장제도와 그렇지 않은 경우의 두 가지로 나뉨)와 민주주의(이 경우 발전, 저발전의 두 가지로 나뉨)을 국민의 건강수준을 설명할 수 있는 가설적 조건으로 채택하는 경우, 각각이 독립변수로서 국민건강수준(종속변수)에 미치는 영향을 살펴보는 것이 아니라, 각 조건이 결합되어 어떻게 요인으로 작용하는지 분석하는 것이다.

퍼지셋 질적비교연구방법은 요인이 되는 조건들을 '속성(attributes)'으로 설명하는데, 구체적으로 속성이 K개일 때, 인과적 결합요인은 수리적으로 다음과 같이 2k개까지 구성될 수 있다 (H는 높은 수준의 의료보장제도를 의미하고, h는 낮은 수준의 의료보장제도를 의미한다. D는 발전된 민주주의를 의미하고, 'd'는 저발전된 민주주의를 나타낸다).

X1= 좋은 의료보장제도가 있고 민주주의의 발전수준이 높음(HD),

X2= 좋은 의료보장제도가 있고 민주주의의 발전수준이 낮음(Hd),

X3= 낮은 수준의 의료보장제도가 있고 민주주의의 발전수준이 높음(hD),

X4= 낮은 수준의 의료보장제도가 있고 민주주의의 발전수준이 낮음(hd)

인과관계를 설명하는 등식은 질적 연구를 기반으로 하여 사례에 '그리고(logical and)'와 '혹은(logical or)'을 적용하는 것으로 발전시킬 수 있다. '그리고(logical and)'는 둘 이상의 조건이 결합되는 복합집합(compound set)을 나타낸다. 등식에서는 곱하기부호(*)로 표기된다. '혹은(logical or)' 은 합집합을 뜻하고 등식에서 덧셈부호(+)로 표기된다.

만약 연구자가 이론적 바탕에 따라 선별한 12개의 사례(국가)가 있는데 그 중 8개의 국가에서 국민들의 평균 건강수준이 높다고 가정하자. 연구자들은 이 8개의 사례(국민의 건강수준이 높은 8개국)를 분석하여 각각에 해당되는 인과적 결합요인으로 1) 좋은 의료보장제도가 있고 민주주의의 발전수준이 높음(HD), 2) 좋은 의료보장제도가 있고 민주주의의 발전수준이 낮음(Hd), 3) 낮은 수준의 의료보장제도가 있고 민주주의의 발전수준이 높음(hD)의 조건을 발견한 경우, 이는 높은 국민건강수준의 조건으로 3개의 결합요인(configurations)이 있을 수 있다는 것을 의미한다. 즉, 건강한 국민의 조건으로 '좋은 의료보장제도가 있고 민주주의의 발전수준이 높음(HD)' 또는 '좋은 의료보장제도가 있고 민주주의의 발전수준이 낮음(Hd)' 또는 '낮은 수준의 의료보장제도가 있고 민주주의의 발전수준이 높음(hD)'이 원인조건들이 될 수 있는 것이다.

높은 국민건강수준 = HD + Hd + hD

불리언(Boolean) 접근법을 적용하면 위 등식은 아래의 등식과 같이 단순해진다. 아래 등식에 따라 '높은 수준의 의료보장제도' 또는 '낮은 수준의 의료보장제도가 있고 민주주의의 발전수준이 높음(H + hD)'인 경우 개인의 건강수준이 높다는 등식이 성립된다. 즉, 높은 수준의 의료보장제도가 있는 경우 민주주의의 발전수준이 높고 낮음과 상관없이 국민의 높은 건강수준에 대

한 조건이 될 수 있고, 또한 의료보장제도의 수준이 낮아도 민주주의의 발전수준이 높은 경우도 높은 건강수준의 조건이 될 수 있는 것이다.

높은 국민건강수준 = HD + Hd + hD

= H(D + d) + hD

= H + hD

다음으로, 퍼지셋 질적비교연구(Fs/QCA) 접근은 변수보다 사례를 더욱 강조한다. 전통적인 변수중심 분석에서 많은 수의 사례가 합쳐지면서(pooling) 개별 사례의 특수성이 사라지기 때문에 각 사례의 역사적・맥락적 미묘한 차이가 감춰지기 쉽다. 즉, 사례 내에서의 이질성이 쉽게 무시될 수 있다는 것이다. 퍼지셋 질적비교연구(Fs/QCA)의 이점은 개별 사례가 변수로 분해되는 것이 아니라 하나의 총체(a whole)로 분석된다는 것이다.

제시한 예시와 같이 하나의 총체(a whole)로서의 영국은 각각의 결합요인들[예를 들어, 의료보장제도가 있고 민주주의가 발전됨(HD), 의료보장제도가 있고 민주주의가 저발전 됨(Hd), 의료보장제도가 없고 민주주의가 발전됨(hD)에 대한 소속점수(membership score)가 각각 생성된다. 셋째, 퍼지셋 질적비교연구(Fs/QCA)는 사례를 특징짓는 방식과 관련하여 유연성을 허용한다. 위에서 언급한 것과 같이, 각 사례는 의료보장제도나 민주주의의 발전정도와 같은 중요한 요인조건에 대해 소속점수(membership score)를 갖게 된다. 단순한 집합이론(set theory)에서 한 사례는 어떤 집합에 속하거나 속하지 않는 두 가지의 경우만 갖게 되는 반면, 퍼지셋 논리는 소속 정도(membership)의 단계를 가능하게 한다. 퍼지셋 질적비교연구는 사례가 갖는 속성들의 정도의 차이를 인정하는 것이다. 이러한 집합의 소속 정도는 연구자에 의해 결정된다. 예를 들어, 영국의 의료보장제도가 일반집합 이론에 따라, '속하거나' '속하지 않는' 의 이분법적으로 구분된다면, 퍼지셋 논리가 결합되면서 의료보장제도의 발전 정도 또는 정부지출액 등 연구자의 연구목적에 의해 각 조건에 대해 퍼지점수가 매겨지게 되는 것이다. 즉, 연구자는 1[소속(fully in)]과 0[소속되지 않음(fully out)]이라는 질적인 한계점(breaking point)을 정의하고 부분적인 소속(membership)을 가능하게 하는 0에서 1사이의 퍼지 점수(membership scores)를 정하는 것이다. 다시 말해, 퍼지셋 점수 매기기는 ‘세밀하게 쪼개진(fine-grained)’ 연속적인 척도로, 조건의 속성과 관련된 본질적이고 이론적 지식을 활용하여 연구자가 신중하게 눈금을 매기는 (calibrate) 것이다(Ragin 2000). 이러한 눈금매기기(calibration)는 질적 개념을 양적으로 비교 할 수 있게 만든다. 예를 들어 한 국가

가 민주주의 국가로 분류되어야 할지 여부나 국민의 건강수준에 따라 국가를 비교하는 경우 연구자는 이론적 지식을 활용하여 퍼지점수 눈금매기기를 실시하고, 이를 통해 건강수준이라는 질적 개념이 양적으로 비교가능해지는 것이다. 퍼지셋 질적비교연구(Fs/QCA)에서 눈금매기기(calibration), 즉 집합의 소속 정도(membership) 결정 은 개념에 따라 다양할 수 있으며, 연구자의 우선순위에 따라 개념적, 이론적, 역사적, 맥락적 배경을 반영한다(Vis 2007).

이러한 퍼지셋 질적비교연구(Fs/QCA)의 특징은 두 가지 이점을 가진다. 첫째, 퍼지셋 질적비교연구(Fs/QCA)는 질적비교연구의 일반적 과정을 적절하게 반영한다. 또한 둘째로, 신중한 눈금 매기기(calibration)를 통해 양적 비교연구를 가능하게 한다. 셋 째, 퍼지셋/질적 비교연구(Fs/QCA)는 사회과학의 여러 명제들에 내재되어 있는 집합이론적(set theoretic) 특성을 반영함으로써 이론과 경험적 자료의 분석이 일치될 수 있도록 한다(Ragin 2000). 퍼지셋 방법론에서 눈금매기기(calibration)는 퍼지점수표(membership scoring scheme)이다. 이는 연구자가 개발한 기준으로, 측정값을 바로 해석할 수 있도록 만든다. 자연과학에서 예를 들면 20C는 0에서 100사이에 있기 때문에 해석할 수 있는 것이다. 예를 들어, "민주주의의 발전은 국민의 높은 건강수준을 위해 필수적이다"는 이론적 서술에서 국민의 높은 건강수준은 민주주의의 부분집합이 된다. 다시 말하면 민주주의는 국민건강의 필요조건이 된다. 이에 대해, Ragin은 집합 관계가 언어적 서술을 구성한 단위이며, 이론에서 묘사된 집합 관계는 양적연구에서는 대개 원인변수의 순효과(net effect) 평가를 중심으로 하는, 변수들 간의 상관관계에 대한 가설로 전환되기 때문에 이론적 담론으로부터 멀어진다고 설명하였다(Ragin 2007).

집합이론(set theory)의 인과관계를 알아보기 위하여 원인(Xi) 으로 상정된 조건 혹은 속성의 소속(membership) 값은 결과(Yi) 의 소속(membership) 값과 동일한 방식으로 계산될 필요가 있다(Ragin 2000). 한 조건의 값이 일관되게 결과의 값보다 높을 때 이를 필요조건이라 하고(Yi ≤ Xi) 한 조건의 값이 결과의 값 보다 항상 낮을 때 이는 충분조건이다(Xi ≤ Yi). 조건이 충분조건인지 필요조건인지 가늠하기 위하여 연구자들은 위 등식에 대하여 각 사례의 H(좋은 의료보장제도)와 D(민주주의) 조건의 퍼지 점수(membership score)를 계산해야 한다. H(좋은 의료보장제도)과 D(민주주의)의 퍼지점수(membership score)가 국민건강수준의 퍼지점수(membership score)보다 일관적으로 낮은 경우, 이 조건은 충분조건이 된다.

예를 들어, 국민의 건강수준이 높다고 평가된 8개 국가는 H(강한 의료보장제도)과 D(민주주의)에 대하여 서로 다른 퍼지점수 (membership score)를 받을 것이다. 만약 싱가폴의 경우 D(강한 의료보장제도)가 0.7, D(민주주의)가 0.5, C(국민건강수준)가 0.8이라면, 인과관계식의 최대치는 0.7이며, 이것이 결과 점수 0.8보다 낮기 때문에 덴마크 사례는 '높은 수준의 의료보장제도’ 또는 ’낮은 수준의 의료보장제도가 있고 민주주의의 발전수준이 높음(H + hD)'이 높은 국민건강수준의 충분조건이라는 주장을 뒷받침한다. 만약 모든 8개 국가가 일관된 결과를 보인다면(Xi ≤ Yi), '높은 수준의 의료보장제도’ 또는 ’낮은 수준의 의료보장제도가 있고 민주주의의 발전수준이 높음(H +

hD)'의 조건은 높은 국민건강수준의 충분조건이다. 앞서 설명한 퍼지셋 질적비교연구의 기본 단계는 크게 다음의 5단계로 요약할 수 있다. (1) 이상형(ideal type)의 특성이나 원인조건을 사례에 대한 질 적 분석과 기존 이론의 지식을 바탕으로 선택한다. (2) 가능한 조건들의 결합(configurative)을 제시한다. 이때, 불리언(Boolean) 접근법을 적용하여 이를 단순화시킬 수 있다. (3) 점수표(측정눈금(calibration))는 사례에 대한 실증적인 지식 과 관련 이론을 기반으로 만들어진다. 연구자를 이를 바탕으로 질적 기준점(break points)이 설정 한다 (4) 각 조건이나 조건 결합(configurative)에 대한 각 사례의 퍼지점수(membership score)가 산출된다.(5) 원인조건의 퍼지점수(membership score)와 필요·충분조건을 가늠하기 위한 결과의 퍼지점수(membership score)가 산출하여 비교한다 (이승윤 2014).

퍼지셋 질적비교연구(Fs/QCA) 는 질적 분석과 양적 분석 모두의 장점을 포함시키기 위한 시도로 개발된 방법론으로. 제대로 수행되는 경우 두 방법론적 사이의 가교 역할을 할 수 있다. 그렇지만 연구자들은 퍼지셋 질적비교연구(Fs/QCA)만의 독특한 기능을 정확히 파악하고 적합한 연구에 적용하는 것이 선차적으로 중요하다.

Reference

이승윤 (2014). 퍼지셋 질적비교연구방법론의 이론과 적용: Fs/QCA 입문, 고려대학교출판부.

Lee, SY. (2013). Fuzzy-set method in comparative social policy: a critical introduction and review of the applications of the fuzzy-set method. Quality and Quantity. 47(4): 1905-1922

Ragin, C. (2008). Redesigning social inquiry: Fuzzy sets and beyond. Chicago: University of Chicago Press.

Ragin, C. (2008). User's guide to fuzzy-set/qualitative comparative analysis.

Ragin, C. (2007). Qualitative comparative analysis using fuzzy-sets (fsQCA). In: B. Rihoux and C. Ragin (eds) Configurational comparative analysis. California: Sage Publications.

Ragin, C. (2006). Set relations in social research: Evaluating their consistency and coverage. Political Analysis, 14(3), 291-310.

Ragin, C. (2000). Fuzzy-set social science. Chicago: University of Chicago Press

Ragin, C. (1991). Introduction: The problem of balancing discourse on cases and variables in comparative social science. Dharwar, India: Sage Publications, Inc.

Ragin, C. (1987). The comparative method: Moving beyond qualitative and quantitative methods. California: University of California Press

Smithson, M. (2005). Fuzzy set inclusion linking fuzzy set methods with mainstream techniques. Sociological Methods & Research, 33(4), 431-461.

Verkuilen, J. (2005). Assigning membership in a fuzzy set analysis. Sociological Methods & Research, 33(4), 462-496.
